# Clinical indications and acquisition protocol for the use of dynamic contrast-enhanced MRI in head and neck cancer squamous cell carcinoma: recommendations from an expert panel

**DOI:** 10.1186/s13244-022-01317-1

**Published:** 2022-12-17

**Authors:** Valeria Romeo, Arnaldo Stanzione, Lorenzo Ugga, Renato Cuocolo, Sirio Cocozza, Mario Quarantelli, Sanjeev Chawla, Davide Farina, Xavier Golay, Geoff Parker, Amita Shukla-Dave, Harriet Thoeny, Antonello Vidiri, Arturo Brunetti, Katarina Surlan-Popovic, Sotirios Bisdas

**Affiliations:** 1 Department of Advanced Biomedical Sciences, University of Naples “Federico II”, Naples, Italy; 2 Department of Clinical Medicine and Surgery, University of Naples “Federico II”, Naples, Italy; 3 Interdepartmental Research Center on Management and Innovation in Healthcare - CIRMIS, University of Naples Federico II, Naples, Italy; 4 Biostructure and Bioimaging Institute, National Research Council, Naples, Italy; 5 Department of Radiology, Perelman School of Medicine, the University of Pennsylvania, Philadelphia, PA USA; 6 Department of Medical and Surgical Specialties, Radiological Sciences, and Public Health, University of Brescia, Brescia, Italy; 7 Department of Brain Repair and Rehabilitation, UCL Queen Square Institute of Neurology, University College London, London, UK; 8 Lysholm Department of Neuroradiology, The National Hospital for Neurology and Neurosurgery, University College Hospitals NHS Trust, London, UK; 9 Department of Computer Science, Centre for Medical Image Computing, Queen Square Institute of Neurology, University College London, London, UK; 10 Department of Radiology, Memorial Sloan Kettering Cancer Center, New York, NY USA; 11 Departments of Medical Physics, Memorial Sloan Kettering Cancer Center, New York, NY USA; 12 Department of Radiology, Cantonal Hospital Fribourg, University of Fribourg, Fribourg, Switzerland; 13 Department of Radiology and Diagnostic Imaging, IRCCS Regina Elena National Cancer Institute, Rome, Italy; 14 Department of Neuroradiology, University Clinical Centre, Ljubljana, Slovenia

**Keywords:** Magnetic resonance imaging, Evidence-based medicine, Squamous cell carcinoma of the head and neck

## Abstract

**Background:**

The clinical role of perfusion-weighted MRI (PWI) in head and neck squamous cell carcinoma (HNSCC) remains to be defined. The aim of this study was to provide evidence-based recommendations for the use of PWI sequence in HNSCC with regard to clinical indications and acquisition parameters.

**Methods:**

Public databases were searched, and selected papers evaluated applying the Oxford criteria 2011. A questionnaire was prepared including statements on clinical indications of PWI as well as its acquisition technique and submitted to selected panelists who worked in anonymity using a modified Delphi approach. Each panelist was asked to rate each statement using a 7-point Likert scale (1 = strongly disagree, 7 = strongly agree). Statements with scores equal or inferior to 5 assigned by at least two panelists were revised and re-submitted for the subsequent Delphi round to reach a final consensus.

**Results:**

Two Delphi rounds were conducted. The final questionnaire consisted of 6 statements on clinical indications of PWI and 9 statements on the acquisition technique of PWI. Four of 19 (21%) statements obtained scores equal or inferior to 5 by two panelists, all dealing with clinical indications. The Delphi process was considered concluded as reasons entered by panelists for lower scores were mainly related to the lack of robust evidence, so that no further modifications were suggested.

**Conclusions:**

Evidence-based recommendations on the use of PWI have been provided by an independent panel of experts worldwide, encouraging a standardized use of PWI across university and research centers to produce more robust evidence.

**Supplementary Information:**

The online version contains supplementary material available at 10.1186/s13244-022-01317-1.

## Key Points

- The clinical role of PWI in HNSCC still has to be defined.
- Evidence-based recommendations are provided for the acquisition of PWI sequence in HNSCC.
- A modified-Delphi approach was used to reach a consensus among selected experts.

**Supplementary Information:**

The online version contains supplementary material available at 10.1186/s13244-022-01317-1.

## Background

Head and neck squamous cell carcinoma (HNSCC) represents the most common malignancy in the highly heterogeneous group of neoplasms affecting this anatomical region. HNSCC management is often challenging since treatment goals include not only survival improvement, but also organ function preservation [1]. While multiple imaging modalities have a recognized role in the assessment of the disease, MRI thanks to its excellent soft tissue contrast offers exquisite anatomical details crucial for accurate diagnosis, staging and follow-up of HNSCC [2, 3]. Moreover, advanced MRI sequences can provide valuable functional information on tumor biology and are increasingly being included in MR protocols not only for lesion characterization in the head and neck region, but also for treatment monitoring of HNSCC [4, 5]. Among these, perfusion weighted MR imaging (PWI) allows to assess cancer tissue vascular properties and has the potential to significantly improve the accuracy of MRI in HNSCC evaluation both for diagnosis and prognosis [6]. Dynamic contrast enhanced (DCE) MRI, based on the serial acquisition of multiple T1-weighted images before, during and after the intravenous injection of a Gd-based contrast agent, is the most frequently used perfusion technique for H&N region, though other perfusion techniques such as dynamic susceptibility contrast (DSC) and arterial spin labeling (ASL) have been proposed [7–9]. The rationale for using PWI in HNSCCs evaluation lies in the possibility of evaluating tumor neoangiogenesis. Indeed, malignant neoplasms induce the formation of new vessels which are characterized by high tortuosity, density, and a variable degree of permeability. Furthermore, PWI can depict areas of tumor hypoxia which could reduce the response to chemotherapy and radiation therapy, both treatments requiring an adequate blood and oxygen supply to the tumor to be effective. Therefore, PWI might be helpful in the early assessment of therapy outcome.

Despite these premises, the role of this technique still remains to be defined in the clinical practice. Furthermore, to obtain reliable and reproducible perfusion parameters, standardization of the acquisition protocol is needed, and minimum technical requirements should be established. To fill the gap of recommendations for PWI technique and its clinical use in HNSCC management, we performed a systematic review of the literature and recruited an international panel of independent experts in the field, aiming to reach a consensus on the most crucial aspects of the technique using a modified Delphi approach, under the auspices of the European Society of Neuroradiology. This initiative is particularly intended to provide clinical indications of PWI to clinicians, radiologists, engineers/physicists, and technicians, as well as to suggest optimal acquisition parameters of a standard PWI protocol in daily clinical practice.

## Methods

### Systematic literature and evidence evaluation

A systematic review of the literature was performed to identify experimental studies on the use of PWI in head and neck cancer over the last 10 years. Different databases, including PubMed, Scopus, and Web of Science, were searched by two researchers with 5 and 8 years of experience. The string search along with inclusion and exclusion criteria is reported in Additional file 1: S1. Selected papers were evaluated by five expert researchers with 8 to 10 years of experience, supervised by a senior researcher with more than 20 years of experience, applying the Oxford criteria 2011 [10], which also allow to adjust the final classification considering additional parameters such as “study quality”, “imprecision”, “indirectness”, “inconsistency”, “small/large effect size”. Each study was then assigned a category from 1 to 5, where “1” is the highest level of quality and “5” the lowest one.

### Questionnaire development

A multidisciplinary local expert team was built, including radiologists, physicists, medical oncologists, surgeons, and radiation oncologists to identify the relevant clinical scenarios in HNSCC patients. A questionnaire was then prepared including several statements, organized in two different categories: “Clinical indications” and “Acquisition protocol”. Details on the included information are reported in Additional file 1: S2.

For both sections, the highest-quality papers were selected according to the level of evidence and were consulted for drafting a set of statements for each topic. The final questionnaire included a total of 20 statements, of which 6 related to clinical indications of PWI and 14 to the acquisition technique.

### Expert panel selection

According to the Delphi procedure [11], a panel of 8 experts was identified comprising of radiologists, scientists, physicists and engineers. Panelists were selected among experts in the field, based on published papers and related research activities.

### Delphi rounds

A modified Delphi approach was used for reaching a final consensus. The questionnaire was sent via email with no physical meeting was arranged. All panelists worked in anonymity. The first questionnaire was then sent via e-mail to panelists who agreed to join this initiative. Panelists were asked to rate the level of agreement for each statement using a Likert scale provided in the questionnaire. A score was therefore assigned from 1 (strongly disagree) to 7 (strongly agree); definitions of agreement scale and recommendations are reported in Additional file 1: S3 and S4, respectively. Panelists were also asked to provide comments, particularly for statements rated with score equal or inferior to 5 (“agree with amendments”). Statements for which scores equal or inferior to 5 were assigned by at least two panelists were modified. Final questionnaires were also externally reviewed by two additional independent experts. A cost effectiveness analysis was also considered for questionnaire building.

## Results

### First Delphi round

After the first round, 9/20 statements (45%), of which 6/6 belonging to clinical indications (100%) and 3/14 (21%) to acquisition protocol sections, were rated with scores equal or inferior to 5 by at least two panelists, and therefore modified according to panelists’ comments. A statement related to the acquisition technique section was also removed as the reported information had been included in a related modified statement. Thus, a total of 19 statements were included in the revised questionnaire.

### Second Delphi round

**Fig. 1:**
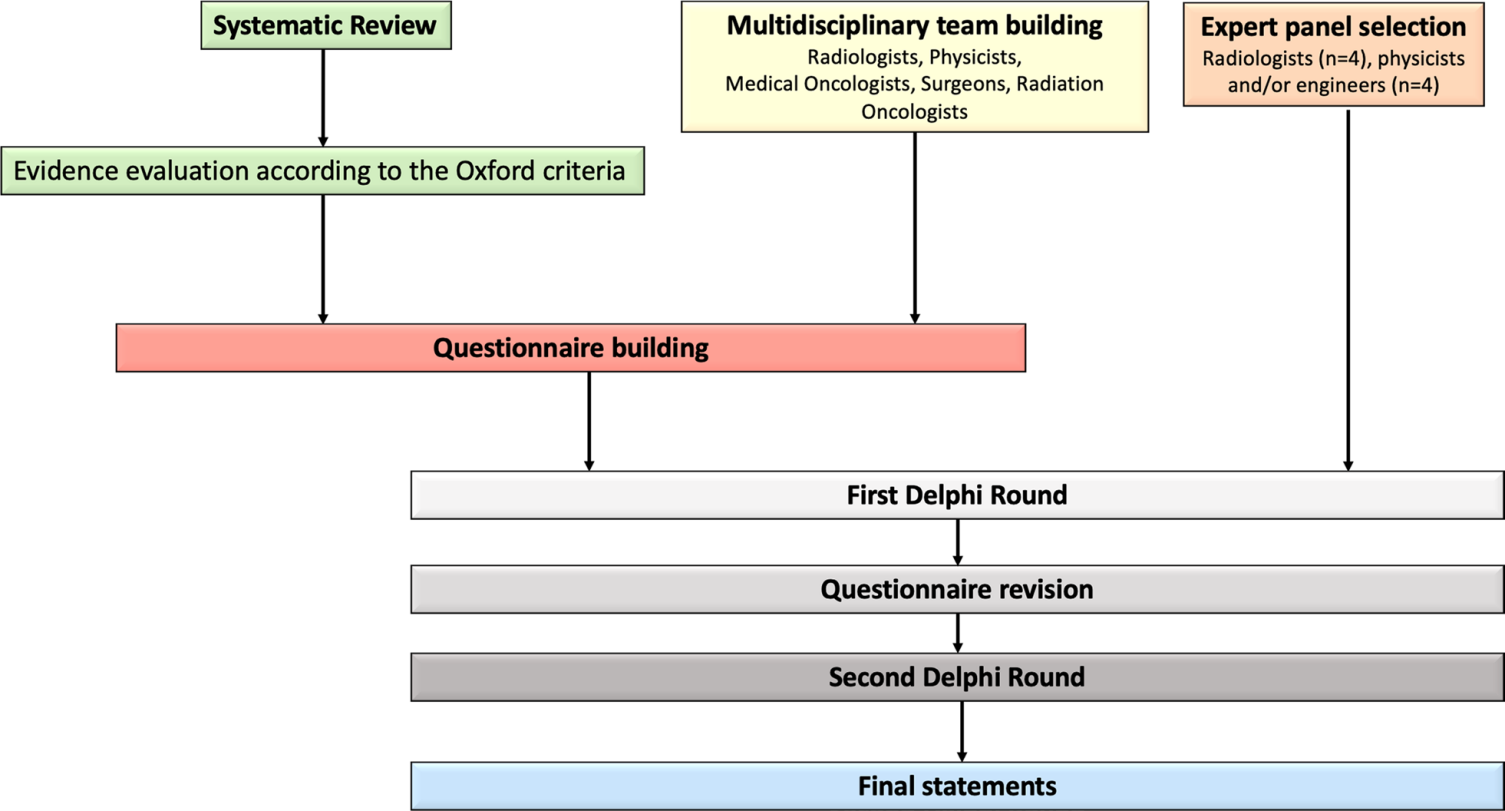
Flowchart illustrating the process of recommendations development

After the second round, two panelists were no longer available. Thus, questionnaires completed by rest of six panelists were collected. Based on the assigned scores, 4/19 (21%) statements obtained scores equal or inferior to 5 by two panelists, all dealing with clinical indications. Based on panelists’ comments, the Delphi process was considered concluded as reasons entered by panelists for lower scores were mainly related to the lack of robust evidence, so that no further modifications were suggested. Issues related on the availability of the PWI sequence among centers and particularly of post-processing imaging software were also considered when considering the final recommendations. Recommendations for clinical indications and acquisition protocol of PWI in HNSCC are reported in Tables 1 and 2, respectively, while the flowchart of all process is illustrated in Fig. 1. The cost-effectiveness analysis could not be performed due to the lack of Level-1 evidence (i.e., randomized clinical trials, meta-analyses) supporting the usefulness of PWI in the clinical practice. Further details are reported in Additional file 1: S5.

**Table 1:** Clinical indications of PWI in HNSCC

Clinical scenario	Recommendation
Diagnosis	May be recommended
N staging	Not recommended*
Radiotherapy planning—gross tumor volume and organs at risk delineation	May be recommended
Early assessment of response during chemo-radiotherapy	Not recommended*
Assessment of response after chemo-radiation therapy/induction chemotherapy	May be recommended*
Detection of local tumor recurrence during follow-up after treatment	May be recommended*

*Statements with lower level of agreements

**Table 2:** Acquisition technique recommendations for PWI in HNSCC

**Perfusion type**	
1. DCE perfusion, achieved with 3D Spoiled-Gradient Echo T1-weighted sequences, should be used	
2. Limited evidence exist on the applicability of DSC and ASL perfusion techniques in the clinical setting	
**DCE acquisition parameters**	
1. Axial images should be acquired on scanners with a field strength of 1.5 or 3.0 Tesla using head and neck or neurovascular coils, with the following minimum parameters	
i. Slice thickness: ≤ 4 mm (recommended: 3 mm)	
ii. Gap: no gap	
iii. In-plane resolution: ≤ 2 × 2 mm	
iv. Fat suppression	
2. T1 mapping sequences with multiple flip angles (suggested not inferior to 3, ideally ranging from 5° to 30°) should be acquired, with the same geometry of DCE, prior to contrast administration to obtain accurate data kinetic fitting	
3. Temporal resolution should be ≤ 5 s, with 5 acquisitions prior to contrast agent administration and a total acquisition time of at least 5 min	
**Contrast agent**	
1. Gadolinium-based contrast agents should be employed at the recommended dosage based on molecule choice with an injection rate ≥ 2 mL/s, followed by at least 20 mL saline flush	
**Image analysis**	
1. Bi-compartimental and extended Tofts models are the current reference standard for quantitative perfusion parameter calculation	
2. The arterial input function should be measured placing a ROI in the carotid arteries (external/internal or common based on lesion location and best time-intensity curve morphology). The use of a population-based AIF can be considered	
**Quantitative DCE parameters calculation**	
1. ROIs should be placed on DCE post-contrast images avoiding areas of hemorrhage, necrosis, cystic components and neighboring vessels and pasted on perfusion maps to extract the quantitative parameters

## Discussion

In this initiative, we aimed to provide evidence-based recommendations for the use of PWI in HNSCC. Based on the results of the Delphi procedure, it emerged clearly that, at present, robust evidence to support the use of PWI in clinical practice is still lacking. Indeed, the majority (80%) of statements in which a full agreement could not be reached were related to clinical indications. Promising clinical applications of PWI are represented by lesion characterization, RT planning, assessment of treatment response during or after chemo-radiation therapy/induction chemotherapy, prediction of therapy outcome and detection of local tumor recurrence during follow-up after treatment. However, evidence with higher quality (multicenter, well-designed prospective studies and clinical trials) is needed to suggest the application of this advanced imaging technique in the assessment of head and neck tumors. Interestingly, some discrepancies were found between the results of the systematic literature search and experts’ opinion. Indeed, while most of the published studies supported the use of PWI for different clinical scopes, i.e., prediction/assessment of the response to treatment, some concerns on its real applicability were raised by the panelists. This divergence in the viewpoint clearly reflects a gap between research and clinical practice, for which more consistent proofs are needed to ensure the transition into clinical routine. On the other hand, the experts were more optimistic on some applications. Despite the availability of limited evidence for specific issues, such as RT planning and assessment of disease recurrence during follow-up, the possible use of PWI was advocated by panelists, thus producing a mild recommendation. Even considering issues related to costs of PWI post-processing software and the time needed for image post-processing and PWI parameters calculation, statements on the mandatory use of PWI could not be formulated. In this light, it appears even more important to provide clear recommendations on acquisition parameters and quantitative parameters calculation, so that robust and reliable scientific findings can be obtained and validated with standardized and well-defined methods, comparable across centers. Each clinical indication and acquisition technique statement is discussed below.

## Clinical indications

### Statement 1. Diagnosis: May be recommended

Most panelists agree that the role of PWI for the diagnosis of HNSCC might not be crucial. Usually, the diagnosis and characterization of HNSCC lesions is made using morphological sequences such as T2w and conventional post-contrast T1w images. Experts suggest a possible role of PWI to differentiate HNSCCs from lymphomas, given that significant differences were found between the two entities in terms of quantitative and semiquantitative PWI parameters [12]. PWI was found to have a possible role to non-invasively characterize tumor microenvironment, with consequential implications on tumor grade, T stage and treatment planning [13–15]. Indeed, PWI parameters and corresponding maps may have a role in defining tumor microcirculation properties such as vessel size and distribution, flow heterogeneity, thus resulting in the spatial assessment of tissue permeability and hypoxia [16, 17]. In several studies, a significant correlation was found between quantitative PWI parameters (K^trans^, Kep and Ve) and tumor stage [18, 19]. An increase of PWI parametric maps heterogeneity was also shown with the progression of T and N stage [18]. PWI-derived histogram analysis parameters were proven to correlate with VEGF and EGFR expression, particularly in oropharynx carcinoma [20, 21] and also in HNSCC nodal metastasis [22]. Nevertheless, the routine use of PWI in clinical practice for the diagnosis of HNSCC could not be endorsed.

### Statement 2. N staging: Not recommended

Several studies in the scientific literature support a possible role of PWI for lymph node (LN) characterization in HNSCC. Indeed, PWI parameters were found elevated in malignant LN compared to the benign ones, independently of short-axis diameter [23]. PWI parameters also negatively correlate with ^18^F-FMISO uptake in malignant LNs, supporting the hypothesis that hypoxic metastatic LNs are poorly perfused [24]. Differences in terms of K^trans^ extracted from pathological LNs in nasopharynx HNSCC were also observed among patients with different N stages, with N3 patients showing the highest values [25]. Conversely, lower K^trans^ values calculated for primary HNSCC of the oral cavity were found in advanced N stage cases, possibly indicating that hypoxic tumors are more prone to nodal dissemination [26]. Despite published studies are limited mainly by small sample size, the proposed recommendation by the local panel for this statement was “May be recommended”. However, panelists scores were concordant suggesting a “downgrade” of such a recommendation toward “Not recommended”, particularly claiming that, at present, there is no proof of a clear benefit of using PWI over PET/CT or ultrasound-guided fine needle aspiration biopsy. Despite the applied changes, there was still a disagreement of two panelists based on the lack of sufficient evidence. Therefore, comparative studies and cost-effectiveness analysis are required to demonstrate the actual potential of PWI in this clinical scenario.

### Statement 3. Radiotherapy planning—gross tumor volume and organs at risk delineation: May be recommended

Based on the current literature, there is no established single imaging modality for adequately defining a RT boosting target volume [27]. Due to the lack of robust evidence on this matter, the recommendation originally proposed was “Not recommended”. Nevertheless, PWI might be useful for RT planning, as suggested in the literature and acknowledged by the experts. Indeed, PWI can identify intratumoral areas of hypoxia, which is one of the most important prognostic factors determining RT failure [28]. In this setting, artificial intelligence (AI)-based models applied to DCE-MRI were built to identify poorly perfused subvolumes of tumors [29, 30]. Considering that RT planning is often made on the basis of different MR images, including both anatomical and functional (e.g., diffusion MR imaging); the recommendation was changed to “May be recommended” after the first round of the Delphi process, achieving a wider consensus among panelists during the second round.

### Statement 4. Early assessment of response during chemo-radiotherapy: Not recommended

Several studies promote the use of PWI for early assessment of treatment response during chemo-RT. A recent systematic review by Bernstein et al*.* including six studies found that K^trans^ and its related histogram analytical parameters were predictive of good clinical outcomes [31]. The majority of available studies report that higher pre-treatment K^trans^ values, that decrease early during chemo-RT, are associated with better treatment and survival outcomes [32–36]. To date, PWI parameters have been applied in prospective clinical trials for the assessment of treatment response [34, 37]. However, some discrepancies are reported in the literature as pre-treatment PWI parameters were unable to predict treatment failures [38], and difference between good-responders and poor/non-responders [39]. In line with these contradicting findings and considering the scores < 5 given by two panelists, the final indication was “Not recommended”. Further standardization of acquisition techniques and quantitative parameters calculation is needed to further promote the use of quantitative PWI in clinical practice, thus allowing to assess the usefulness of the routine performance of a contrast-enhanced MRI during treatment that could influence treatment strategy.

### Statement 5. Assessment of response after chemo-radiation therapy/induction chemotherapy: May be recommended

Like the early assessment of the response to treatment, most of the available evidence suggest a possible role of quantitative PWI parameters for the assessment of treatment response. Baseline values of Ve were found to be higher in responders compared to non-responders oral cancer patients, reflecting an increase of the extravascular-extracellular space due to cell death [40]. In the same way, nasopharyngeal carcinoma patients showing complete response exhibited lower post-treatment K^trans^ values and larger delta K^trans^ between pre- and post-treatment MRI examinations [41]. Considering the promising findings of the literature, the recommendation proposed by the local team was “Recommended”, that was successively downgraded to “May be recommended” during the Delphi procedure due to the lack of randomized clinical trials and concerns on parameters variability across centers. This position was further highlighted by the lower level of agreement on this statement, as two experts still assigned scores ≤ 5 due to the lack of robust evidence.

### Statement 6. Detection of local tumor recurrence during follow-up after treatment: May be recommended

Most of the studies conducted on the usefulness of PWI for the discrimination of post-treatment changes and local recurrence are based on the use of semiquantitative parameters and DSC-PWI [9, 42, 43]. For this statements, opinion among the panelists was divided as some of them advocated the use of PWI for imaging follow-up while others were skeptical based on the limited number of studies published so far [44]. A final “May be recommended” option was finally assigned as the discrepancy was mainly due to the lack of available evidence and considering the need to further promote research activities in this field.

## Acquisition protocol

The first two statements were not modified after the first Delphi round. Indeed, the majority of panelists agreed that DCE-MRI obtained with 3D-spoiledgradient- echo T1-weighted images is a choice of sequence for acquiring DCE-MRI data [31, 45], due to shorter acquisition time and high contrast-resolution of the head and neck region. It should be noted that new generation sequences are expected to enter the clinical arena in the near future, based on radial acquisition technique, which is less prone to artefacts caused by voluntary/involuntary motions [46].

Regarding the application of DSC and ASL perfusion techniques, preliminary studies have demonstrated a correlation between tumor blood flow as estimated by ASL and DCE-MRI techniques [47, 48] and pathological degree of tumor differentiation [49]. However, experts agreed that limited evidence exist to promote the use of DSC and ASL techniques in clinical practice.

Statement 3 includes recommendations on basic acquisition parameters. Here, the aim is to provide general advice, to be adjusted based on MRI scanner, patients and lesions’ features. In detail, it is advisable that slice thickness remains under 4 mm, with the optimal value of 3 mm, with no gap and an in-plane resolution < 2 × 2 mm^2^. Use of fat suppression methods is also recommended. Dedicated head and neck phased array coils should be used, taking the possibility of also employing surface coils into account, to improve image details and signal-to-noise ratio (SNR). Drawbacks of the use of surface coils are related to the low depth of penetration, thus limiting their use to superficial organs, such as the larynx. Since information on the in-plane resolution was given, it was decided not to include recommendation on FOV, as it is usually prescribed depending on lesion size and position.

Flip angle should also be kept as low as possible, due to the short TR, and T1 mapping with variable flip angle should also be performed, to calculate the baseline T1, thus allowing a more accurate estimation of T1 changes related to contrast agent injection, as reported in statement 4.

Expert agreed that temporal resolution of the PWI sequence should be kept high (i.e., lower than 5 s) by using parallel imaging, without compromising SNR and spatial resolution. This is crucial to accurately calculate the transfer of contrast agent through capillary vessels and thus assess vascular function [50]. The use of a power injector is also essential, with a rate ≥ 2 ml/s, (ranging in the literature between 2 and 4 ml/s), acknowledged as the most widely adopted in literature.

Different pharmacokinetic models were used, with the most extensively being used as proposed by Tofts and co-investigators [51], enabling the estimation of all the most widely used parameters, such as K^trans^, Kep, Ve and Vp. The measurement of AIF, probing the amount of contrast agent entering the tissue of interest, is necessary for the estimation of quantitative parameters. Usually, a region of interest (ROI) is placed on a major vessel near the tumor site for AIF calculation, that is internal/external carotid artery for the head and neck region. As the reliability of AIF measurement is challenging due to partial volume effects, temporal resolution-related undersampling, and inflow effects, panelists also suggested the possibility of using a population-based AIF to improve precision, especially when temporal resolution is sub-optimal. While the aim of this initiative was to provide recommendations for PWI acquisition technique, a statement was also included on quantitative parameters calculation from DCE images, as several concerns were raised recently in the literature on their variability. As multiparametric maps are computed from DCE-MR images, ROI delineation should be performed on native post-contrast perfusion images, preferably the subtracted ones, using T2w and high-resolution post-contrast T1w images to better delineate tumor margins. While drawing ROIs, care should be taken to exclude areas of necrosis, hemorrhage, cysts and neighboring vessels. No specific instructions on which post-contrast time point should be used for tumor segmentation can be derived from the literature at this stage. However, considering the enhancement pattern of HNSCCs, the calculation of quantitative parameters should not be influenced by the time point at which tumor segmentation is performed. Nonetheless, this issue remains to be clarified.

## Conclusions

Evidence-based recommendations on the use of PWI have been provided by an independent panel of experts worldwide, combining information derived from the current literature with experts’ opinion using the Delphi technique. Based on this experience, there is still no sufficient evidence and resources to promote the use of PWI in clinical practice. Nevertheless, a wider consensus was reached among panelists on acquisition parameters to be used to standardize the acquisition of the PWI sequence and computation of corresponding quantitative parameters. Experts further encourage a standardized use of PWI across research and clinical centers to produce more robust evidence, preferably by way of multicenter/multiplatform studies in order to assess the clinical value of this challenging and promising functional MR technique for future applications in head and neck.

## Recommendations’ update

The provided recommendations will be updated every two years based on the availability of relevant evidence on the matter.

## Supplementary Information

**Additional file 1**. string search; inclusion/exclusion criteria; information included in the PWI questionnaire; definition of the agreement to a 7-scores Likert scale; definitions of recommendations; and cost-effectiveness analysis.

## Data Availability

Data can be provided by the corresponding author under reasonable question.

## Abbreviations

AIF: Arterial input function
ASL: Arterial spin labeling
DCE: Dynamic contrast enhanced
DSC: Dynamic susceptibility contrast
FOV: Field of view
HNSCC: Head and neck squamous cell carcinoma
K^trans^: Volume transfer constant
LN: Lymph node
PWI: Perfusion weighted imaging
RT: Radiotherapy
SNR: Signal-to-noise ratio
Ve: Volume fraction of extravascular extracellular space
Vp: Volume fraction of plasma space
