# Supplementary material for: Clinical indications and acquisition protocol for the use of dynamic contrast-enhanced MRI in head and neck cancer squamous cell carcinoma: recommendations from an expert panel
Source: Insights Imaging. 2022 Dec 17;13:198. doi: 10.1186/s13244-022-01317-1 (PMC9759606; doi:10.1186/s13244-022-01317-1)
Supplement: Supplementary file 1 — Additional file 1. string search; inclusion/exclusion criteria; information included in the PWI questionnaire; definition of the agreement to a 7-scores Likert scale; definitions of recommendations; and cost-effectiveness analysis. [file 13244_2022_1317_MOESM1_ESM.pdf]

ELECTRONIC SUPPLEMENTARY MATERIAL

Clinical indications and acquisition protocol for the use of dynamic contrast-enhanced MRI in head and neck cancer squamous cell carcinoma: recommendations from an expert panel

S1. String search and inclusion/exclusion criteria

("Head and Neck" OR "Head and neck" OR "head and neck" OR “H&N” OR “H-N” OR “HN” [title/abstract] OR Head and Neck Neoplasms [MeSH]) AND (MR OR MRI OR magnetic resonance imaging [title/abstract] or Magnetic Resonance Imaging [MeSH]) AND ("PWI" OR "PW" OR "Perfusion-Weighted" OR "Perfusion Weighted" OR "Perfusion-weighted" OR "Perfusion weighted" OR "perfusion-weighted" OR "perfusion weighted" OR “DCE-MRI” OR "DCE" OR "Dynamic Contrast-Enhanced" OR "Dynamic Contrast Enhanced" OR "Dynamic contrast-enhanced" OR "Dynamic contrast enhanced" OR "dynamic contrast-enhanced" OR "dynamic contrast enhanced" [title/abstract] AND (“2004/01/01”[Date - Publication] : “2019/01/01”[Date - Publication]) AND “english”[Language])

Inclusion criteria were as follows: 1) studies dealing with HNSCC; 2) systematic reviews and metanalysis; 3) experimental studies conducted on human subjects; 4) DCE-MRI sequence acquired for the calculation of quantitative parameters. In vitro studies, case reports, editorials, articles dealing with tumors other than HNSCC (e.g., salivary glands, esophageal cancer) or not dealing with cancer nor imaging were excluded.

S2. Information included in the PWI questionnaire.

For the “Clinical indications” section, selected papers were organized into sub-categories according to the dealt topic, i.e., tumor characterization, staging, assessment/prediction of the response to chemo-radiotherapy (RT), prediction of patients’ prognosis and detection of tumor recurrence. For the DCE-MRI “Acquisition protocol” section, the following information was extracted from each published paper: imaging window (pre, during or post-treatment period), type of DCE-MRI sequence, pharmacokinetic model applied, field strength (1.5 or 3T) of MRI scanner, type of radiofrequency coil, repeating time (TR)/echo time (TE), flip angle, matrix size, field of view (FOV), voxel size, slice thickness, number of slices, inter-slice gap, T1 mapping sequence, when performed, , temporal resolution, acquisition time, arterial input function (AIF) modality, type of gadolinium based contrast agent with contrast dose and injection rate. Invitations to join the expert panel were sent via email on October 2019. Revised questionnaires were sent to panelists in June 2020. All feedbacks were collected by September 2020.

S3. Definitions of the agreement according to a 7-scores Likert scale.

1: strongly disagree

2: disagree

3: moderately disagree

4: major amendments needed to reach agreement

5: minor amendments needed to reach agreement

6: agree, with suggestions

7: strongly agree, no amendments needed

S4. Definitions of recommendations

Mandatory: current evidence strongly support the use of the sequence and therefore its routine use in the clinical setting is endorsed/advocated.

Recommended: current evidence are sufficient to support the use of the sequence in the clinical setting and therefore it should be acquired although not mandatory.

May be recommended: current evidence show the usefulness of the sequence, but further studies are necessary to support its use in the clinical setting.

Not recommended: current evidence are insufficient and/or heterogeneous. Hence, the use of the sequence is not recommended in the clinical settings.

S5. Cost-effectiveness analysis

Due to the lack of Level-1 evidence (i.e., randomized clinical trials, metanalyses) supporting the usefulness of PWI in the clinical practice, a cost-effectiveness analysis could not be performed. Despite the fact that the sequence could easily be installed on clinical MRI scanners, dedicated and possibly costly software, as well as support by specific professional figures such as engineers/physicists, are required to assist radiologists for image post-processing and quantitative estimation of parameters. Furthermore, the procedure for PWI parameters extraction can be quite time-consuming and thus not consistent with a routine clinical practice. Such factors have been taken into account when formulating the final recommendations.
